# Isolation of *Phaeobacter* sp. from Larvae of Atlantic Bonito (*Sarda sarda*) in a Mesocosmos Unit, and Its Use for the Rearing of European Seabass Larvae (*Dicentrarchus labrax* L.)

**DOI:** 10.3390/microorganisms9010128

**Published:** 2021-01-08

**Authors:** Pavlos Makridis, Fotini Kokou, Christos Bournakas, Nikos Papandroulakis, Elena Sarropoulou

**Affiliations:** 1 Department of Biology, University of Patras, Rio 26504 Patras, Greece; bio3235@upnet.gr; 2 Aquaculture and Fisheries Group, Department of Animal Sciences, Wageningen University and Research, 6700AH Wageningen, The Netherlands; fotini.kokou@wur.nl; 3 Biotechnology, and Aquaculture, Hellenic Center for Marine Research, Institute of Marine Biology, P.O. Box 2214, 71003 Heraklion Crete, Greece; npap@hcmr.gr (N.P.); sarris@hcmr.gr (E.S.)

**Keywords:** larviculture, fish microbiota, antagonism, microbiology, challenge test

## Abstract

The target of this study was to use indigenous probiotic bacteria in the rearing of seabass larvae. A *Phaeobacter* sp. strain isolated from bonito yolk-sac larvae (*Sarda sarda*) and identified by amplification of 16S rDNA showed in vitro inhibition against *Vibrio anguillarum.* This *Phaeobacter* sp. strain was used in the rearing of seabass larvae (*Dicentrarchus labrax* L.) in a large-scale trial. The survival of seabass after 60 days of rearing and the specific growth rate at the late exponential growth phase were significantly higher in the treatment receiving probiotics (*p* < 0.05). Microbial community richness as determined by denaturing gradient gel electrophoresis (DGGE) showed an increase in bacterial diversity with fish development. Changes associated with the administration of probiotics were observed 11 and 18 days after hatching but were not apparent after probiotic administration stopped. In a small challenge experiment, seabass larvae from probiotic treatment showed increased survival (*p* < 0.05) after experimental infection with a mild pathogen (*Vibrio harveyi*). Overall, our results showed that the use of an indigenous probiotic strain had a beneficial impact on larval rearing in industry-like conditions.

## 1. Introduction

Most marine fish larvae during the first weeks after hatching feed on zooplanktonic organisms. Rearing of marine fish larvae is based worldwide on the use of two mass-produced zooplanktonic organisms, namely rotifers, *Brachionus* sp., and brine shrimp *Artemia* sp. [1].The mass culture of live feed results in increased numbers of opportunistic bacteria, which are easily transferred to the larval tanks [2]. During the first days of feeding, there is a huge increase in bacterial abundance in the larval gut [1]. In the yolk-sac stage, the microbiota of the larvae is aerobic and highly diverse, while after feeding starts, there is a shift to an anaerobic microbiota [3].

During the period of feeding with live feed, gut microbiota in marine fish is dominated by γ-proteobacteria (Vibrionaceae, Pseudomonadaceae, and Oceanospirillaceae are among the most abundant families) [4]. Later, during the development of the digestive system, gut microbiota becomes more stable and is characterized by the dominance of *Vibrio*, *Pseudomonas*, *Acinetobacter*, *Corynebacterium*, *Alteromonas*, *Flavobacterium*, and *Micrococcus* species [5].

The rearing of marine fish larvae in commercial hatcheries suffers often from high mortalities, which could be due to nutritional deficiencies of cultured live food, zootechnical imperfections of the rearing system, or pathogenic microorganisms such as opportunistic Vibrios. *Vibrio anguillarum* is a common pathogen in aquaculture and it has been reported as a cause of mortalities in the rearing of marine fish larvae [6]. One approach to limit the growth of opportunistic bacteria is the use of probiotic bacteria, which may inhibit opportunists, either by indirect antagonism, such as competition for nutrients, iron, attachment sites, or by direct antagonism through the production of bacteriocins [7]. An example of bacteria producing such compounds is *Phaeobacter* spp., which commonly appear in larviculture systems [8]. These bacteria produce tropodithietic acid, a compound that inhibits Vibrios [9]. Production of tropodithietic acid is accompanied by the production of a brown pigment, which results in brown colonies in Marine Agar and makes easier their identification in Petri dishes. Probiotic bacteria, in addition to being antagonists for harmful bacteria, promote the health of fish larvae through stimulation of the immune system by triggering increased lysozyme level, enhancement of complement activity, or upregulation of pro-inflammatory cytokines [10]. An important requirement for the use of a bacterial strain as a probiotic in aquaculture is that it is possible to culture the probiotic bacteria in advance and provide them to the larvae through the diet or in the water of the rearing tanks. Probiotic bacteria of the Roseobacter group have been repeatedly reported in the literature for their positive effect during the rearing of cold-water marine fish species. Nevertheless, they have been tested only in small-scale trials during the first weeks of rearing.

Atlantic bonito (*Sarda sarda*) is a small tuna species common in the Mediterranean, and it was used as a secondary species in the project SELFDOTT, “From capture based to self-sustained aquaculture and domestication in bluefin tuna, *Thunnus thynnus*” with the target to produce juveniles of this fish species for stock enhancement [11].

This study aimed to use an indigenous strain of *Phaeobacter* sp. isolated from yolk-sac larvae of Atlantic bonito (*Sarda sarda*) in the rearing of seabass (*Dicentrarchus labrax* L.) larvae and to determine the effect of the addition of probiotic bacteria after 60 days of rearing in a large-scale trial. This would allow us to evaluate the effect of probiotics after completion of metamorphosis and transition to the juvenile stage in seabass. Besides, we wanted to evaluate changes in fish microbiota during the critical first weeks of the rearing of seabass and to determine a possible effect of protection against pathogens in a small-scale challenge test with *Vibrio harveyi*.

## 2. Materials and Methods

### 2.1. Rearing of Bonito Larvae

About 20,000 yolk-sac larvae of Atlantic bonito (*Sarda sarda*) were obtained from broodstock at Instituto Español de Oceanografia (Mazarrόn, Spain) and were stocked at the facilities of Hellenic Center for Marine Research in Crete, Greece in a 40 m^3^ tank filled with nonfiltered seawater (salinity 40 g/L) at 23 °C, dissolved oxygen 7.1 mg/L, and pH 8.3. Borehole water (salinity 35 g/L and 20 ± 1 °C) was used as inflow water thereafter. The water temperature throughout the rearing was 21.5 ± 0.5 °C, dissolved oxygen 7.2 ± 0.5 mg/L, and pH between 7.7 and 8.3. The rate of water renewal increased progressively throughout the experiment.

Microalgae, *Chlorella minutissima* (Foti and Novak), were added daily in the tank until 12 DAH (days after hatching). Feeding was based on the daily administration of enriched rotifers (3 DAH), newly hatched *Artemia* sp. nauplii (from 3 DAH), gilthead seabream (*Sparus aurata*) yolk-sac larvae (from 10 DAH), seabream eggs (from 12 DAH), enriched Artemia metanauplii (12–15 DAH), frozen seabream eggs (from 15 DAH), minced fish (from 20 DAH), and artificial diet (from 15 DAH). The population density of rotifers and *Artemia* sp. metanauplii in the tank was maintained at 1.5 and 0.2 ind./mL, respectively. The mesocosm tank also showed inert productivity of zooplanktonic organisms (ciliates, tintinids, copepods) that could potentially contribute as live prey items for the fish larvae.

### 2.2. Microbiology Sampling of Bonito Larvae

Samples of five larvae were taken 0, 7, 14, and 20 DAH and were anesthetized with 3-aminobenzoic acid ethyl ester 0.2 mg/mL (0.2% MS-222, Sigma). The larvae were washed once with 50 mL sterile seawater through a mesh with 250 μm pore size, were subsequently individually homogenized, and then plated after serial 10-fold dilutions on Marine Agar plates (Difco Laboratories, Detroit, MI, USA). The plates were incubated for 7 days at 20 °C in the dark and colonies were counted after one, two, and seven days. Frequently encountered, similar-in-appearance colonies were considered dominant and were selected from all sampling days from marine agar plates, pure-cultured, and thereafter stored by use of a MicrobankTM Freezer kit (PRO-LAB Diagnostics, Richmond Hill, Canada) at −80 °C.

### 2.3. PCR Amplification

Bacterial isolates were later regenerated in test tubes with marine broth and thereafter spread on Marine Agar to evaluate the purity of the cultures. Bacterial cells (1:20) were heated in distilled water at 98 °C for 15 min and centrifuged for 10 min according to Jensen, Bergh, Enger, and Hjeltnes [12]. The universal bacterial primers 27f and 1492r were used to amplify the 16S ribosomal genes [13]. PCR was performed in 50 μL reaction mixtures comprising 0.05U *Taq* polymerase (Promega, Madison, WI, USA), 2.5 mM MgCl_2_, 1× buffer, 200 μM dNTPs, 1 μL diluted cell suspension, and 0.5 μM of each of the 27f and 1492r primers. Reactions were carried out in a PCT-200 thermal cycler (MJ Research Inc., Watertown, MA, USA), with an initial denaturation step of 95 °C of 15 min followed by 30 cycles of 92 °C for 1 min, 55 °C for 1 min, and 72 °C for 45 s; the final extension step was performed at 72 °C for 5 min. PCR products were purified using a DNA purification kit (Qiagen, DNeasy Tissue Kit, Venlo, The Netherlands), quantified by a NanoDrop spectrophotometer, and visualized by 1% agarose/TAE gel electrophoresis.

### 2.4. DNA Sequencing and Construction of a Phylogenetic Tree

PCR products were excised from the gel with sterile razor blades and purified applying Nucleospin (R) Extract II (Macherey Nagel, Düren, GmBh, Düren, Germany). The concentration of purified PCR products was estimated using a NanoDrop ND-1000 spectrophotometer (NanoDrop Technologies, Wilmington, DE, USA) and cycle-sequenced in both directions with Big Dye Terminator Ver. 3.1 (Applied Biosystems Inc., ABI, Foster City, CA, USA). The sequences were electrophoresed on an ABI 3130 × l DNA sequencer (Applied Biosystems Inc.). Sequences were vector and quality trimmed before downstream analysis. Forward and reverse sequence reads were compared, resulting in a total of 24 sequences. Obtained sequences were searched against the NCBI database rRNA_typestrains/prokaryotic_16S ribosomal RNA and the nr database by BLASTn search (https://www.ncbi.nlm.nih.gov/) for annotation, as well as against the silva high-quality ribosomal RNA database. Multiple sequence alignments were carried out using a SINA 1.2.11 sequence alignment tool with default parameters [14]. A phylogenetic tree was generated including one nearest neighbor applying maximum likelihood analysis implemented in MEGA 7 with default parameters. All positions containing gaps and missing data were eliminated, resulting in a total of 181 positions in the final dataset. The tree was also computed by RA × ML with the GTR model for tree computation and the Gamma rage model for likelihoods [15,16]. Bootstrap replicates were set to 1000. The evolutionary history was inferred by applying two tree computational approaches. First, by using the maximum likelihood method based on the Tamura–Nei model [16], initial tree(s) for the heuristic search were obtained automatically by applying Neighbor-Join and BioNJ algorithms to a matrix of pairwise distances estimated using the maximum composite likelihood (MCL) approach and then selecting the topology with the superior log likelihood value.

### 2.5. Inhibition Tests

The isolate BON02 was tested for in vitro antagonism against three *Vibrio anguillarum* fish pathogenic strains: LMG 4437, isolated from Atlantic cod (*Gadus morhua* L.) by Dr. J. Bagge; LMG 10862, serovar 2, isolated from Atlantic cod by J.L. Larsen; and LMG 10863, serovar 3, isolated from rainbow trout (*Oncorhynchus mykiss*) by J.L. Larsen. The isolates originated from the Belgian Co-ordinated Collections of Micro-organisms (https://bccm.belspo.be). These three bacterial strains and BON02 were cultured in 5 mL of Marine Broth 2216 (Difco Laboratories, Detroit, MI, USA) at 20 °C. The culture period was 24 h for the pathogens and 10 days for BON02. A modification of a well-diffused agar assay of [17] was used in this study. From the liquid culture, an aliquot of 50 μL was spread on a Marine Agar petri dish, a hole was made by use of the tip of a sterile glass Pasteur pipette at the center of the dish, and 5 μL of the BON02 culture was transferred to the hole. The plates were incubated in the dark at 20 °C, and the diameter of the inhibition zone was measured after one week. Three replicate Petri dishes were used for each pathogen tested.

### 2.6. Rearing of Seabass Larvae

Seabass eggs were transferred to eight 500 L tanks at 100 eggs/L stocking density. The tanks were connected pairwise to a 1 m^3^ biofilter system, which removed ammonia from the water through a recirculation system. Four tanks contained the treatment receiving probiotic bacteria and four tanks contained the control treatment. The eggs and yolk-sac larvae were incubated in total darkness until 8 DAH (days after hatching). The photoperiod was 12L:12D thereafter until 60 DAH at a light intensity of 40–60 lux. Surface film removers were operated in each tank from 1 to 14 DAH, which removed the surface oil film to facilitate the filling of the swim bladder. The average fish length and wet weight in each tank were measured daily, and twice per week, respectively.

Feeding with rotifers, *Brachionus* sp. started 8 DAH. At the same time, microalgae *Chlorella minutissima* (Foti and Novak) were added to a concentration of 25 × 10^4^ cells/mL. At 14 DAH, we started feeding with enriched Artemia metanauplii, whereas feeding with an artificial diet started at 32 DAH. An automated feeding system was used for both live food and artificial diet to avoid problems with cannibalism among seabass larvae.

The water temperature increased gradually after hatching from 16 °C to 20 °C throughout the rearing trial. Bioencapsulation of probiotic bacteria through live food was done three times a week, and through this process, probiotic bacteria were given four times in total to the larvae via rotifers and four times via Artemia. In these cases, live food was added manually to both treatments. In all other instances, food was added to the tanks through an automated system [18].

Fish growth was monitored in all tanks during the whole experiment. At each sampling point, 10 fish were taken from each tank and the fish length was measured after inspection through a stereoscope.

### 2.7. Bioencapsulation of Probiotic Bacteria

The bacterial strain BON02, which was identified by 16 rDNA sequencing as *Phaeobacter* sp., was inoculated in 2 L liquid cultures of Marine Broth 2216 (Difco Laboratories) in Erlenmeyer flasks with no agitation for 4–5 days at room temperature. At the end of the culture period, the color of the culture was checked to verify antibiotic production in the culture.

Probiotic bacteria were delivered to seabass larvae (*Dicentrarchus labrax* L.) after bioencapsulation in live food [19]. Probiotic bacteria were harvested by centrifugation at 3000 rpm for 20 min. Cultures of rotifers (100 rotifers/mL) and *Artemia* (50 *Artemia*/mL) were incubated in suspensions of bacteria in seawater at a concentration of 5 × 10^7^ bacteria/mL for 30 min. At the end of the incubation period, live food organisms were rinsed through a mesh and fed to the larvae. Throughout the experiment, probiotics were provided to the larval rearing system three times a week starting from 11 DAH during the first weeks after hatching.

### 2.8. Challenge Test

A small-scale challenge test was run with seabass larvae at 30 DAH, where larvae of both treatments were transferred to a 1 L beaker (30 specimens in each beaker). In one group, a mild bacterial pathogen *Vibrio harveyi* VH2 [20] was added at 10^5^/mL final concentration, whereas in a control group, no pathogen was added. The test was run in three replicates for all possible combinations. The larvae were kept unfed in an incubator at 20 °C and mild aeration was added to each beaker. Mortalities were monitored and dead larvae were registered and removed.

### 2.9. Microbiological Sampling of Seabass Larvae

Ten larvae were sampled for microbiological analysis from each tank 4, 11, 18, 25, and 32 DAH. After transfer from each tank, the larvae were anesthetized with MS-222, washed with 50 mL sterile seawater, and homogenized in 5 mL sterile seawater in a glass homogenizer. Serial 10-fold dilutions were plated on 90 mm Petri dishes of Marine Agar (Difco Laboratories) and TCBS (thiosulfate-citrate-bile salts-sucrose) agar. The Petri dishes were thereafter incubated at room temperature (20–22 °C) for 10 days. A subsample of 1 mL of the homogenate was stored at −80 °C for use in denaturing gradient gel electrophoresis (DGGE) analysis.

Samples were taken as well from live food organisms (rotifers and Artemia) at the end of the bioencapsulation process for microbiological analysis. The live food organisms were rinsed, washed with 50 mL sterile seawater, and 2 mL was homogenized in a glass homogenizer. A subsample of one millimeter of the homogenate was stored at −80 °C for use in denaturing gradient gel electrophoresis (DGGE) analysis.

### 2.10. DNA Extraction and PCR

DNA extraction was performed according to [21], as modified by [22] with several extra modifications. DNA extraction began with the addition of 20 μL of 100 mg/mL fresh lysozyme (final concentration 2 mg/mL) and 1.7 mL lysis buffer to 200 μL of homogenized tissue followed by incubation with rotation at 37 °C for 30 min. Fifty microliters of 20 mg/mL Proteinase K (final concentration 1 mg/mL) and 100 μL of 10% (*w*/*v*) sodium dodecyl sulfate (SDS) (final concentration 0.5% (*w*/*v*)) were added, and the sample was incubated in a circulating water bath at 55 °C for 1 h. The lysate was then extracted with an equal volume of phenol pH 8.0. The aqueous phase was extracted with an equal volume of phenol-chloroform-isoamyl alcohol (25:24:1; pH 8.0), then extracted with an equal volume of chloroform-isoamyl alcohol (24:1). One-tenth volume of 3 M NaOAc (pH 5.3) and 2 volumes of absolute ethanol were added to the aqueous extract. The extracts were kept overnight at −20 °C. Following 15 min centrifugation at 13,000 rpm at 4 °C, the pellet was washed with 1 mL ice-cold 80% ethanol and centrifuged for 5 min at 13,000 rpm at 4 °C. The pellet was air-dried overnight and resuspended in 50 μL *DNase*/*RNase*-free water. DNA yield was quantified by a NanoDrop spectrophotometer and DNA quality was visualized by the agarose gel electrophoresis of 5 μL of DNA extract. DNA extracts were stored at 4 °C until use.

PCR was carried out in a Biometra Tpersonal Thermocycler (Biometra biomedizinische Analytik GmbH, Jena, Germany) under aseptic conditions in a laminar flow chamber to avoid contamination by environmental bacteria. Sterile water has been used as a negative control in all PCR reactions. A ~1500 bp fragment of the bacterial 16S rRNA locus was amplified for DGGE analysis using the bacterial universal primers 27F (5’-AGAGTTTGATC(AC)TGGCTCAG-3’) and 1492R (5’-ACGG(CT)TACCTTGTTACGACTT-3’) [23]. Each PCR reaction (20 μL) contained 0.5 pmol/μL of each primer, 50 ng of template DNA, 1× Green GoTaq Flexi Buffer (Promega), 1.5 mM MgCl_2_, 0.5 units GoTaq Flexi DNA Polymerase (Promega), and 0.25 mM of each dNTP. PCR conditions were as follows: 95 °C for 2 min followed by 30 cycles of 94 °C for 30 s, 52 °C for 30 s, and 72 °C for 90 s plus 1 s per cycle, with a final extension step of 5 min at 72 °C [24]. One microliter of this PCR product was used as template DNA for a nested PCR.

Nested PCR was carried out in a total volume of 50 μL. The ingredients and their concentrations were the same as the previous PCR reaction, apart from the primers that were 357F (5′-CCTACGGGAGGCAGCAG- 3′) and 518R (5′–ATTACCGCGGCTGCTGG-3′) [25]. Primer 357F contained a GC clamp of 40 bp at the 5′ end (5′-CGCCCGCCGCGCGCGGCGG GCGGGGCGGGGGCACGGGGGC-3′), which is needed for DGGE. PCR conditions were as follows: 95 °C for 5 min followed by 10 cycles of 94 °C for 30 s, 55 °C for 30 s, 72 °C for 60 s, and 25 cycles of 92 °C for 30 s, 52 °C for 30 s, and 72 °C for 60 s, with a final step of 10 min at 72 °C. Nested PCR products were run in DGGE.

### 2.11. DGGE Analysis

DGGE was carried out using the polyacrylamide electrophoresis apparatus. Gels were 1 mm thick (17.5 × 15.5 cm^2^) and 8% *w*/*v* polyacrylamide 37.5:1 with a gradient of urea (Merck, Kenilworth, NJ, USA) and formamide (Fluka, Buchs, Switzerland) between 40 and 60%. Gels were poured with a 50 mL volume Gradient Mixer (Biorad, Hercules, CA, USA) and prepared with 1× TAE buffer (pH 8; 40 mM Tris base, 20 mM acetic acid, 1 mM EDTA). Gels were run at 75 V for 19 h at 60 °C in 1×TAE buffer. Polyacrylamide gels were stained with SYBR Gold nucleic acid gel stain (Molecular Probes, Eugene, OR, USA) for 30 min at room temperature under gentle shaking and viewed under UV. Gel images were captured with a Kodak DC120 camera with the appropriate filter. Community richness was estimated as the total number of bands present in a sample, and the Jaccard community similarity was calculated by scoring the presence/absence of each band horizontally in the gel. For the latter, only samples that were run on the same gel were compared. Two-way ANOVA was used to assess the impact of sampling day (development) and probiotic administration on microbial richness, while two-way Permanova was used to evaluate such impact on the microbial composition, using the Jaccard distance as a metric (presence/absence of bands on the gel). The similarity in the microbial composition within each group was assessed with the Jaccard metric.

## 3. Results

### 3.1. Bacterial Strains Isolated from Bonito Larvae

A total of 27 bacterial strains were isolated. Annotation of the retrieved sequences against the nr database of NCBI placed, with an identity range between 95% and 100% and e-values <1.78 × 10^−56^, seven of the isolated bacteria to Vibrionaceae, four to Rhodobacteraceae, three to Pseudoaltermonadaceae, two to Staphylococcaceae, and two to Oceanospirillales. Only one annotation was found for the families Shewanellaceae, Altermonadaceae, Enterobacteriaceae, Flavobacteriaceae, Micrococcaceae, and Moraxellaceae. Interestingly, among the four individuals of Rhodobacteraceae, one bacterial isolate (BON02) was identified as *Phaeobacter* with 99% identity, an e-value of 0, and a bit score of 1391. A BLASTn search against the rRNA_typestrains/prokaryotic_16S ribosomal RNA, as well as against the Silva high-quality ribosomal RNA database, resulted in the same annotations. All sequences obtained in the current study are available in GenBank with accession numbers MK037395 to MK037418.

Liquid culture aliquots of BON02 (*Phaeobacter* sp.) inhibited the growth of all three *Vibrio anguillarum* strains. The inhibition zones had a diameter of 2.8 ± 0.1, 3.4 ± 0.1, and 3.2 ± 0.1 cm for strain LMG4437, LMG10862, and LMG10863, respectively. The strain LMG4437 showed significantly smaller inhibition zones compared with the other two *V. anguillarum* strains (*p* < 0.05).

### 3.2. Phylogenetic Tree

**Figure 1:**
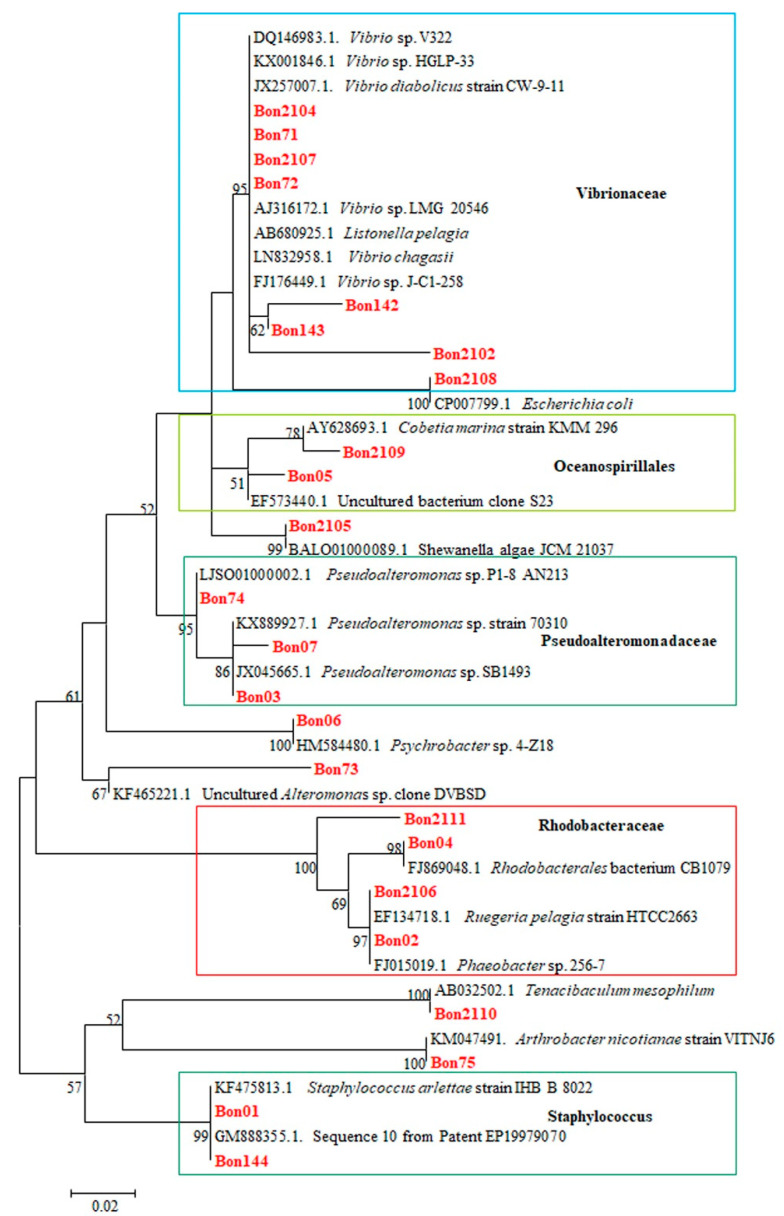
Molecular Phylogenetic analysis by Maximum Likelihood method based on the Tamura-Nei model. The percentage of trees in which the associated taxa clustered together is shown next to the branches. The analysis involved 47 nucleotide sequences. All positions containing gaps and missing data were eliminated. There were a total of 181 positions in the final dataset. Evolutionary analyses were conducted in MEGA7 [2]. In red, samples of the present study are shown. Within the frame in the light blue are representatives of the Vibrionacae, in the light green of the Oceanospirillales, in the olive green of the Pseudoalteromonadaceae, in the red of the Rhodobacteraceae, and in the dark green the representatives of the genus Staphylococcus.

Phylogenetic tree analysis involved 47 nucleotide sequences. The tree with the highest log likelihood (−960.80) is shown (Figure 1).

### 3.3. Experiment with Seabass Larvae

**Figure 2:**
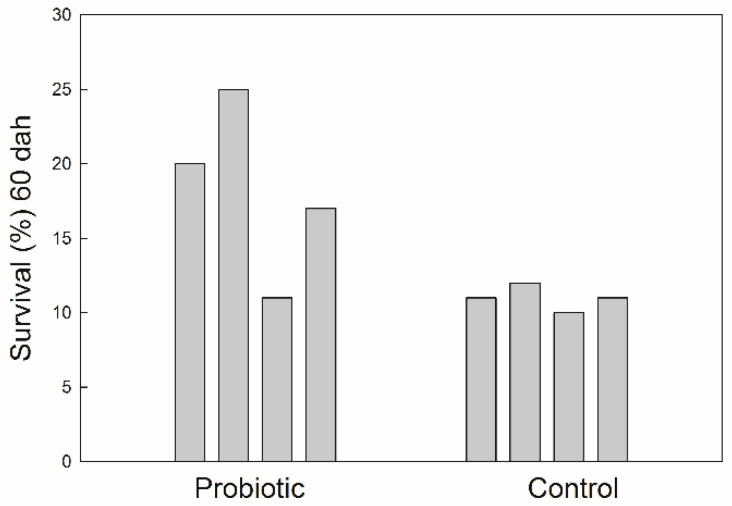
Survival of seabass juveniles expressed as a percentage of numbers of eggs stocked at the beginning of the trial.

**Figure 3:**
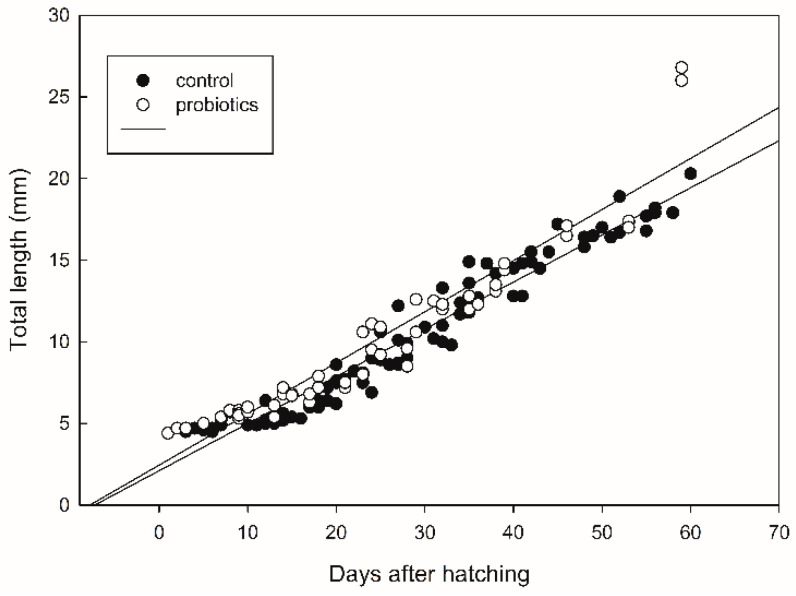
Growth expressed as total fish length (mm) in seabass larvae and juveniles. A regression line was drawn for each treatment. The regression line with the highest slope represents the growth in the probiotic treatment.

The survival of seabass after 60 DAH was significantly higher (*p* < 0.05) in the treatment receiving probiotics (Figure 2). Fish growth, expressed as the fish length, was also influenced by the addition of probiotics. The values of total fish length were log10-transformed and a regression line was drawn for each treatment (Figure 3). There was significant difference in the growth rate (the slope coefficient) between the two treatments (*p* < 0.05), but no statistical difference for the intercept value.

### 3.4. Microbial Community Profiles of the Seabass Larvae

**Figure 4:**
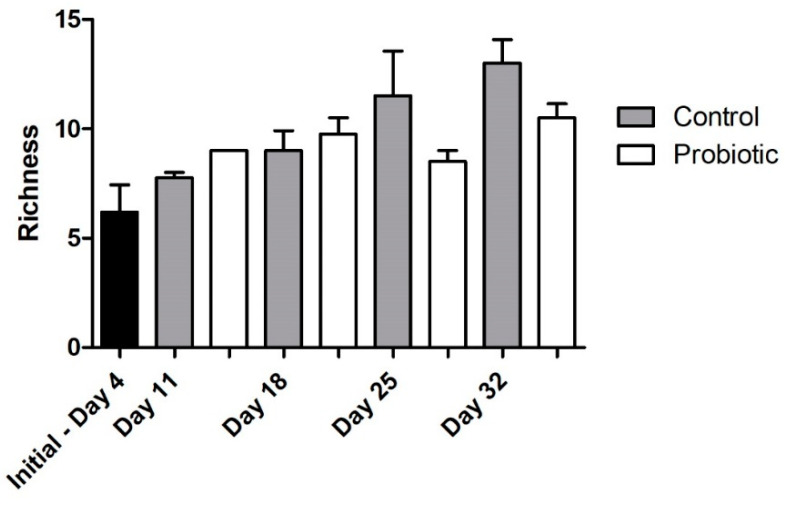
Microbial richness during seabass larvae development as indicated by denaturing gradient gel electrophoresis (DGGE) analysis (bar plots show the number of different bands) between the control and the probiotic treatment. Two-way ANOVA was performed (treatment and time), showing a significant effect of developmental stage (*p* = 0.004), but not probiotic administration in the microbial community over time.

**Figure 5:**
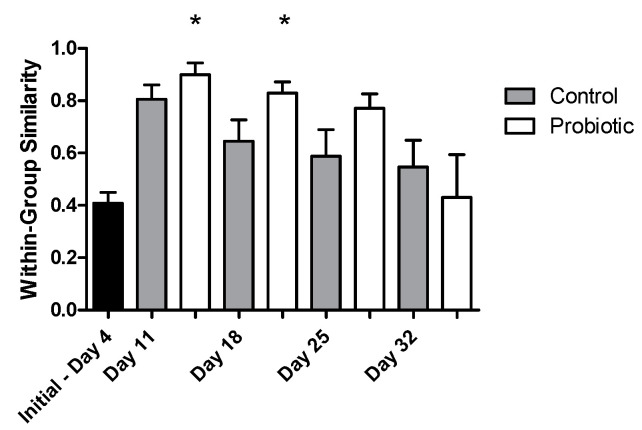
The similarity of the microbial profiles based on the Jaccard metric during seabass larvae development as indicated by DGGE analysis (values closer to 1 indicate a higher similarity between microbial profiles) between the control (grey) and the probiotic (white) treatment. A nonparametric t-test was performed, showing a significant effect (*p* < 0.05) of probiotic administration in the microbial community profiles between days 11 and 18 with the initial day (4 days after hatching (DAH); stars).

Microbial richness, as indicated by DGGE analysis, overall significantly increased over time (two-way ANOVA, *p* = 0.004), starting from a low richness during early larval stages (Figure 4). No significant effect was found, due to the addition of the probiotic strain during larvae development in the number of bacterial taxa (number of bands in DGGE). The similarity of the individuals within each group increased initially from 4 to 11 DAH, but then decreased over time (Jaccard metric for presence/absence; higher similarity when values are closer to 1; Figure 5). Individuals fed with the probiotic had more similar communities compared to the control groups, especially during days 11 and 25 (values closer to 1; *p* < 0.05).

**Figure 6:**
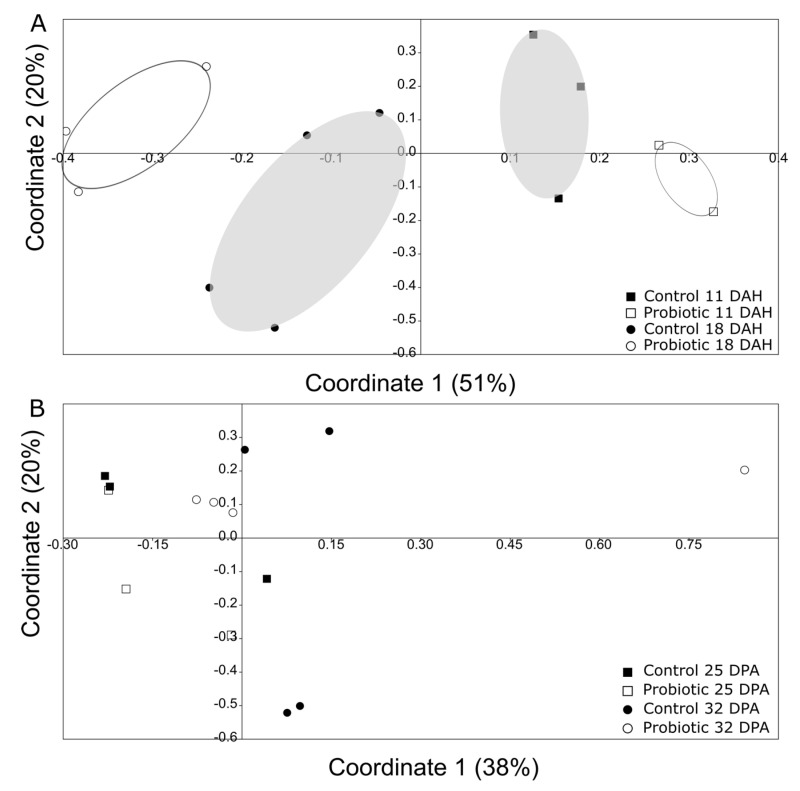
Principal coordinate analysis (PCoA) using Jaccard metric (presence/absence of bands) based on the DGGE information, comparing (**A**) day 11 and 18 in one gel (grey—Control, white—Probiotic), and (**B**) day 25 and 32 in the other gel (grey—Control, white—Probiotic). There was a significant clustering (two-way Permanova, *p* < 0.05; for more information, look at Table 1) based on both the time point and the treatment between days 11 and 18, but not between days 25 and 32.

Comparison of the microbial communities between 11 and 18 DAH revealed a significant clustering of the microbial communities primarily according to time and then to treatment (Figure 6A; two-way Permanova; Table 1). However, such a separation was less clear for days 25 and 32 (Figure 6B).

## 4. Discussion

In this study, we tested the use of an indigenous probiotic bacterial strain, which was isolated from a mesocosm unit at the facilities of the Hellenic Center for Marine Research in Crete, in the rearing of seabass larvae and juveniles in industrial-like conditions. Sequencing of 24 isolated bacterial strains and subsequent annotation and phylogenetic analysis revealed two main groups; the Vibrionaceae (8 bacterial strains) and the Rhodobacteraceae group (four bacterial strains). In a study with tuna larvae reared in different rearing systems, a high individual variability in terms of bacterial community profile as analyzed by the use of PCR-DGGE was observed [26]. Interestingly, the group of Rhodobacteraceae included one strain (BON02), which was identified as *Phaeobacter* sp. The isolated putative probiotic *Phaeobacter* sp. showed inhibitory activity against *Vibrio anguillarum* strains in vitro. In a previous study where it was searched in a turbot farm for strains with inhibitory activity against Vibrionaceae, the majority of Roseobacter (former genus name of Phaeobacter) strains were isolated during spring and early summer [27], as was observed during this study.

Various *Phaeobacter* strains have been isolated from scallop (*Pecten maximus*) [27], larval tank walls [28], tank walls and water from fish tanks, Artemia, rotifers, and zooplankton in a Danish turbot hatchery [29], seawater, biofilms in submerged structures in coastal sites [30], the outlet of fish tanks and Artemia culture tanks, as well as phytoplankton and rotifer cultures from two Greek hatcheries [31]. Nevertheless, it has never been reported to be isolated from marine fish larvae. This is, to our best knowledge, the first report of isolation of *Phaeobacter* sp. from marine fish larvae. It has been shown that these bacteria are unable to colonize the larval gut, but they could colonize the rearing system, such as a tank surface, or a biofilter [32]. It appears that this *Phaeobacter* strain was able to colonize yolk-sac larvae either on the body surface or in the gut before the shift to an anaerobic microbiota. The sampled bonito larvae were not surface-sterilized but only washed with sterile seawater.

*Phaeobacter* isolates have been applied earlier in small-scale trials either bioencapsulated in live food or added as a component of biofilter microbiota. Although we added the probiotics bioencapsulated in live food organisms, it is likely that these bacteria colonized the biofilter and influenced the microbial communities of the larvae through their effect on water microbiota.

Regarding the microbial community richness during the development of the seabass larvae, our analysis using DGGE revealed an increase in the microbial richness over time (Figure 4). Studies related to the microbial community development during the early stages support similar findings [33]. However, as succession proceeded, the bacterial communities were shown to become more similar between individuals (Figure 6B), potentially due to more deterministic factors and less random colonization by different bacterial phylotypes that are known to occur during the early larval stages [5]. The addition of the probiotic on day 11 seemed to affect the bacterial diversity in the gut, which was also observed on day 18 (Figure 6A). Interestingly, there were fewer bacterial phylotypes when probiotics were added to the diets (Figure 4), while the individuals were more similar to each other compared to the control group (Figure 5). This could potentially occur due to the presence of Phaeοbacter in the gut, which can result in inhibition of gut colonization by other bacterial populations.

The addition of probiotics in the rearing of seabass larvae and juveniles had a significant beneficial effect on both survival and growth (Figure 2 and Figure 3). The effect on survival was measured 60 days after hatching, at a period when mass mortality in seabass rearing generally stops, so the effect of probiotics was final. Growth was also higher in the late larval phase compared with the control tanks. It has been shown in many larval experiments that in tanks with a high survival rate with a lot of fish, a lower larval growth rate is expected compared with tanks with few survivors. This has not been shown in our experiment and it indicated that probiotics had a generally positive effect for seabass larvae and juveniles, not only related to the inhibition of pathogenic bacteria.

Single strains of probiotics can hardly work in all kinds of rearing systems for fish larvae and all fish species. Therefore, the application of a commercial product of probiotics that works for all fish species in all kinds of rearing systems somehow seems an illusion. Our study suggests that the use of bacteria isolated from the specific farm that will use these probiotics may have higher chances of success. A similar approach is in one sense the use of “maturated water”, where there is spontaneous recruitment of autochthonous bacterial strains in a biofilter. These bacterial populations have a positive effect on the rearing of larvae, although the identity of the bacteria established and the members that have a positive effect need not be identified. An additional advantage for the use of indigenous bacteria is that there is no biosecurity risk for the farmer, as the probiotics are selected from a group of bacteria already present on the farm.

Previous publications describing the use of *Phaeobacter* probiotic strains in the rearing of marine fish larvae were done for the first days after hatching and on a small experiment scale. It is the first time in this paper that *Phaeobacter* probiotics were tested on such a large scale and the effect on larvae was measured for such a long period (60 DAH), demonstrating the practical significance of the results for commercial aquaculture.

## Figures and Tables

**Table 1:** Two-way Permanova based on the Jaccard distance of the microbial community profiles (indicated by the DGGE analysis) between 11 and 18 DAH.

	Sum of Square	Degrees of Freedom	Mean Square	F	*p*
Time	1.0058	1	1.0058	29.944	0.0001
Treatment	0.18078	1	0.18078	4.5.382	0.0095
Interaction	0.39836	1	0.39836	11.859	0.0001
Residual	0.40308	12	0.03359		
Total	1.988	15			

## Data Availability

The data presented in this study are available on request from the corresponding author. The data are not publicly available due to short time margins.
